# Characterization of Polymeric Microneedle Arrays for Transdermal Drug Delivery

**DOI:** 10.1371/journal.pone.0077289

**Published:** 2013-10-23

**Authors:** Yusuf K. Demir, Zafer Akan, Oya Kerimoglu

**Affiliations:** 1 Department of Pharmaceutical Technology, Marmara University Faculty of Pharmacy, Istanbul, Turkey; 2 Department of Biophysics, Celal Bayar University School of Medicine, Manisa, Turkey; RMIT University, Australia

## Abstract

Microfabrication of dissolvable, swellable, and biodegradable polymeric microneedle arrays (MNs) were extensively investigated based in a nano sensitive fabrication style known as micromilling that is then combined with conventional micromolding technique. The aim of this study was to describe the polymer selection, and optimize formulation compounding parameters for various polymeric MNs. Inverse replication of micromilled master MNs reproduced with polydimethylsiloxane (PDMS), where solid out of plane polymeric MNs were subsequently assembled, and physicochemically characterized. Dissolvable, swellable, and biodegradable MNs were constructed to depth of less than 1 mm with an aspect ratio of 3.6, and 1/2 mm of both inter needle tip and base spacing. Micromolding step also enabled to replicate the MNs very precisely and accurate. Polymeric microneedles (MN) precision was ranging from ±0.18 to ±1.82% for microneedle height, ±0.45 to ±1.42% for base diameter, and ±0.22 to ±0.95% for interbase spacing. Although dissolvable sodium alginate MN showed less physical robustness than biodegradable polylactic-co-glycolic acid MN, their thermogravimetric analysis is of promise for constructing these polymeric types of matrix devices.

## Introduction

Delivering pharmacologically potent molecules to the deeper layers of the skin, in minimally invasive manner, would serve the purpose of effective delivery; pain free, bio safe, patient friendly, self-applicable systems [1].

Transdermal minimally invasive manner means fusion of non-noninvasive (transdermal patches) and invasive systems (hypodermic needle injections), is aimed combining advantages of both systems, and excluding their drawbacks. A great example of this manner is microneedle arrays (MNs) [2].

MNs are novel miniaturized devices, have collections of needles less than 2 mm height on assemble. MNs enable delivery of small, hydrophilic drugs, and also transportation of lipophilic, and macro molecular weight biotherapeutics through microconduits that were physically created by microneedle (MN) while disruption of the stratum corneum (SC), and do not trigger the pain receptors and blood vessels [3]–[5].

There is exhaustive research relating microneedle fabrication techniques [6]–[10], materials [6], [10]–[14], types [15]–[17], geometry [18], [19], and application approaches [13], [20].

Since there is a tremendously growing market for highly accurate micro devices as minimally invasive needles, and miniature molds; microfabricating three-dimensional microstructures is a key factor. For instance, due to being economical, fast, micromachining seems to be the promising method for creating microstructures on various materials [21]–[23]. However, vibrations may negatively affect the precision and surface quality of materials during micro machining. Therefore, reducing the vibrations of machining systems is highly preferable and of critical importance [24] and, that could be eliminated using ultra precision positioning stages [25].

Selecting microneedle type and application approach is of great importance. Due to being economical, bio safe, having advanced functionalized features (such as biocompatible, dissolvable, swellable, and biodegradable), not causing cross contamination, possessing straightforward machinability, and presenting highly accurately repetition in large scale production, polymer materials are sought after drug matrixes, micro fluidic devices, and Lab-on-a-chip systems in biotechnology and medicine [10], . For instance, as opposed to most of the used MNs in the literature, polymeric MNs have specialty in the field of transdermal drug delivery, thus they could be preferred to other type of MNs, such as the brittle and non-biocompatible silicon [17]. In essence, because of their abovementioned features and being patient-specific that discards risk of cross contamination, major interest of this work was selecting polymer materials, and optimizing their fabrication parameters, where they were classified according to their dissolution behavior (e.g., dissolvable, swellable, and degradable behavior).

Dissolvable MNs are promising candidates for facilitating the rapid release of macromolecules. They should have significant robustness for pushing them to the skin, and their fabrication procedure should be organic free solvent, and set at ambient conditions. This strategy ensures that drugs are delivered to specific targets and taken up immediately, which is plausible for short-term applications [1]. Dissolvable MNs were made of maltose [28], carboxymethylcellulose (CMC) [29], amylopectin [30], poly (methylvinylether/maleic anhydride) (PMVE/MA, Gantrez® AN-139) [6], sodium hyaluronate [31], and chondroitin sulphate+dextrin [32].

Swellable materials can be formed by chemically or physically cross-linking polymers. Chemical cross-linking leaves traces of toxic cross linkers, thus limiting its use [33]; therefore physical cross-linking methods are generally preferred for medical applications. Swellable MN could create perpetual micro channels, and enable prolonged delivery when integrated with a drug reservoir. They may also provide bolus and pulsatile delivery if combined with electrical source. In swellable MNs, delivery of macromolecules is governed by cross-link density of the hydrogel that also abolishes the function of other limiting factors, such as SC barrier, and drug encapsulating limitation of the microneedle [34]. Cross linked (PMVE/MA)-poly (ethylene glycol) 10 000 (PEG) [34], and PVA-dextran [14] were produced as hydrogel swelling MNs.

Biodegradable polymers require high temperatures during microfabrication of drug loaded MNs, however, they may damage the incorporated biomolecules. They could be preferred for slow release of active pharmaceutical ingredients (APIs), and heat resistive APIs [14], [35], [36]. Poly lactic-co-glycolic acid (PLGA) [37], polycarbonate [38], poly lactic acid (PLA) [30], poly glycolic acid (PGA) [37], and polystyrene [39] were produced as biodegradable MNs.

In this study, dissolvable sodium alginate (SA) and hydroxypropyl cellulose (HPC) (H and M grades), swellable physically cross-linked polyvinyl alcohol (PVA) and gelatin hydrogels, biodegradable chitosan and PLGA polymers were used to fabricate solid, out-of-plane, polymeric MNs from pyramidal male master templates of the following dimensions: 900-ìm height (700-ìm column height and 200-ìm pyramid tip height), 250-ìm needle base width, 500-ìm interneedle base spacing, and 100-ìm apex diameter [40], [41].

**Figure 1:**
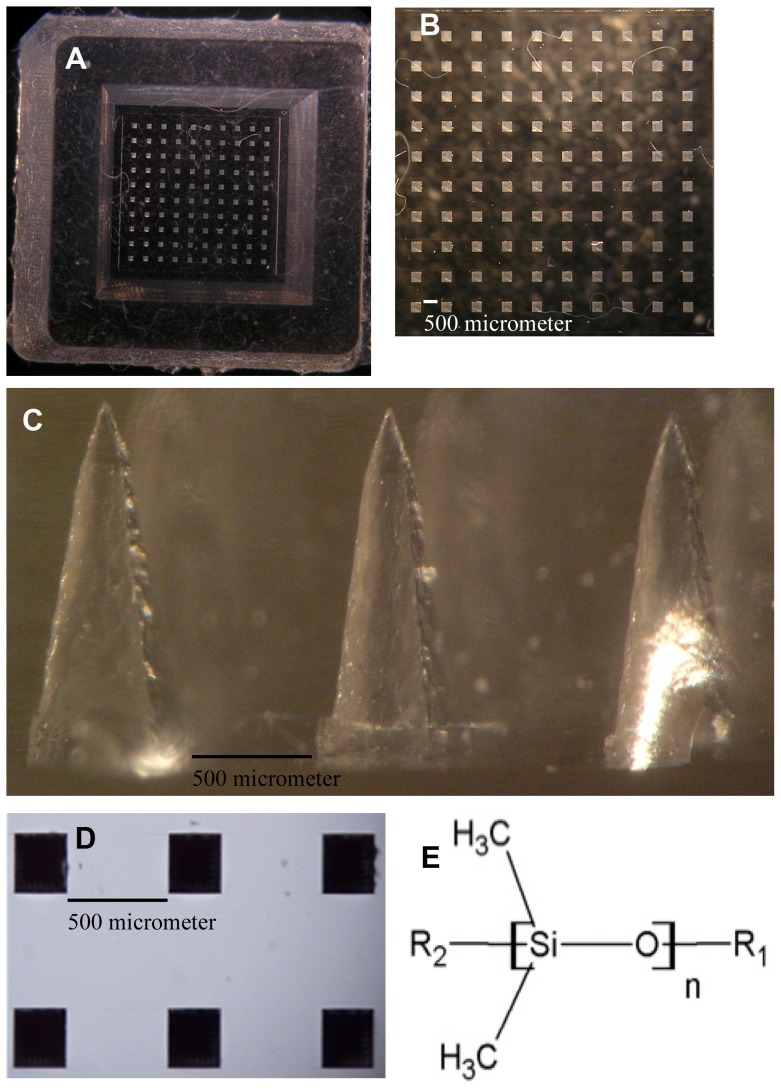
Digital photographs of PDMS micromold parts, and chemical formulas of PDMS. (A) Digital photograph of a 10×10 PDMS micromold fabricated from the pyramidal master template. (B) Top view of the PDMS microholes. (C) Digital representation of the cross-section of the PDMS micromold assembled by conventional micromolding techniques from pyramidal master templates (scanned with an Olympus SZX7 Stereo Microscope; captured using an Olympus C5060 WZ digital camera, Olympus Corporation, Lake Success, NY; and sorted with Adobe® Photoshop® CS5 Extended, Version 12.0×64, Adobe Systems Inc., San Jose, CA). (D) Digital image of a 2×3 needle PDMS micromold. (E) Chemical formulas for polysiloxane and PDMS [46] (Drafted with Chem Sketch Freeware 12; Advanced Chemistry Development, Inc., Ontario, Canada).

Almost all polymeric MN fabrication processes were combined with conventional Polydimethylsiloxane (PDMS) micromolding, due to having unique features. PDMS (Fig. 1(A – (E))) has favorable thermal stability, and low thermal conductivity properties. It is also biologically and chemically compatible, safe, extremely flexible, hydrophobic, non-hygroscopic, and inert under physiological conditions. PDMS is optically transparent and mechanically firm, offering adjustable stiffness and surface adhesion energy [10], [42]–[46].

**Figure 2:**
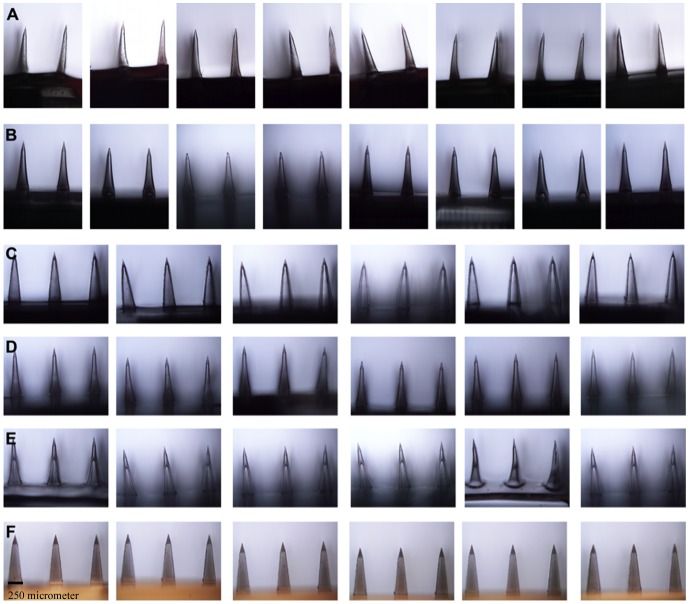
Digital photographs of sections from 10×10 dissolvable MNs fabricated from PDMS micromolds. (A) SA MNs. (B) HPC-M MNs. (C) HPC-H MNs. (D) Cross-linked swellable PVA-gelatin MNs. (E) Chitosan MNs. (F) PLGA MNs.

**Figure 3:**
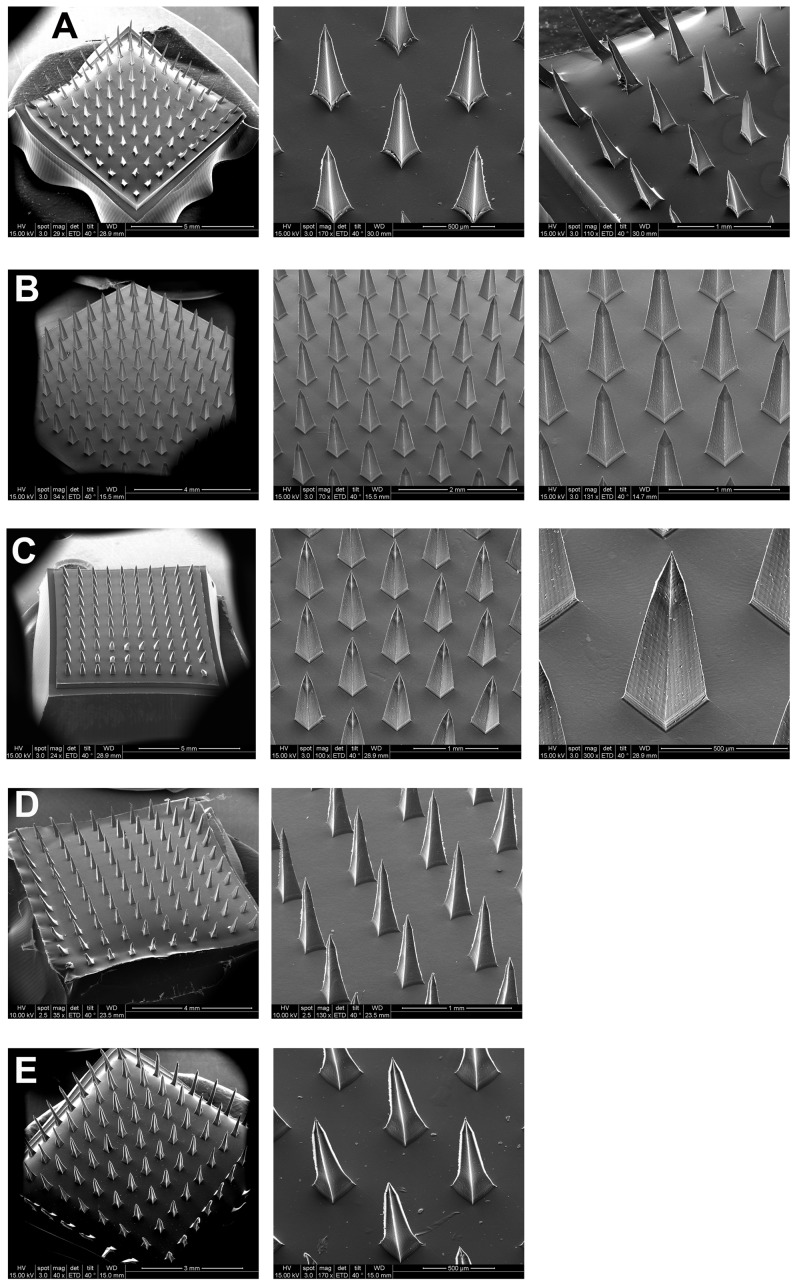
SEM photographs of parts from 10×10 MN arrays. (A)–(E) show polymeric MN arrays that were replicated from the pyramidal master template. (A) SA MNs. (B) HPC-M MNs. (C) HPC-H MNs. (D) Cross-linked PVA-gelatin MNs. (E) Chitosan MNs.

This study utilized a vibration minimized micromachined microdevice with conventional micromolding technology, using the fabrication method and methodology defined for the first time in [22], [23]. Solid out-of-plane pyramidal master templates were used to manufacture PDMS micromolds (Fig. 1(A) – (D)), and micron-scale polymeric needles (Fig. 2(A) – (F), (Fig. 3(A) – (E))) that were inverse copies of male master templates, and perpendicular to the base surface. The resulting microdevices were precisely fabricated, replicable, compatible with micromachining, and reusable even after hundreds of uses.

## Materials and Methods

### PDMS Micromold Manufacturing

This part was carried similar to [22], [23]. A master template, 10×10 pyramidal array of micromilled MNs, was mounted with glue on the center of a 10×10 mm square of Acrylonitrile butadiene styrene (ABS) material. Inverse replicas of master MNs were assembled using 10 g of PDMS (Sylgard; Dow Corning, Midland, Michigan, USA) at a 10∶1 ratio of pre-polymer to curing agent [47]. The PDMS was poured into the ABS material to ensure that the MN array was completely covered. PDMS-filled ABS containers were centrifuged for 20 minutes at 3500 rpm and 5°C to remove air bubbles (Universal 32R; Hettich Zentrifugen, Tuttlingen, Germany).

PDMS-filled ABS containers were cured overnight in an oven (Memmert, Braunschweig, Germany) at 80°C, followed by cooling to ambient temperature. The cured PDMS molds (Fig. 1(A) – (C)) with inverse patterns were negative female molds, and used for the fabrication of male polymeric MNs.

### Physical characterization of polymeric MN arrays

Olympus BX-51 fluorescent microscope (Olympus Corporation, Tokyo, Japan) with image processing and analysis software (BS 200 Pro Image Processing and Analysis Software; BAB Imaging System, Ankara, Turkey) was used for physical characterization of polymeric MNs. The captured image sizes visualized with 640×480 at 10× magnification. The viewfinder (Twain Viewfinder 3.0.1; Pixera Corporation, Los Gatos, CA, USA) mode was set to brightfield light. Interactive geometric measurements were conducted in cases where the scale magnification, scale unit, and yield ratio was 10×, micrometers, 1, respectively.

### Microfabrication of Dissolvable MN Arrays

Not only PDMS mold production but also fabrication of water-soluble MNs were followed from [22], [23] in order to create desired microneedle shapes and arrays, where they have prepared through the micromilling and elastomer molding approaches. Mainly here, spin-casting approach was then applied [22] to fabricate water-soluble MNs. The optimized concentrations of polymer solutions were prepared (explained below). 90 mg of each polymer solution/gel was injected into each PDMS micromold using a 1-ml, sterile, disposable needle-free syringe (Hayat Tibbi Aletler, Istanbul, Turkey). Filled PDMS molds were gently placed in the ABS pyramidal molds, and the ABS lids were screwed on. To spread the gel over the entire MN base and fabricate consistent, well-defined 10×10 pyramidal arrays, centrifugation at 3500 rpm for 20 minutes was applied (Universal 32R; Hettich Zentrifugen, Tuttlingen, Germany). Afterward, the MNs were dried for 24 hours under ambient conditions [40].

To prepare aqueous blends of the sodium alginate (SA) solution, the required amount of Fluka alginic acid sodium salt derived from brown algae was gently sprinkled over cold ultrapure Milli-Q water. The suspension was mixed with a spatula until it formed a homogenous mixture, and 90 mg of 10% (w/w) SA gel was injected into the PDMS micromold according to the above procedure.

During water soluble HPC gel preparation, the rate of polymer hydration was accelerated by adding dry powder to eight times its weight of water at a temperature of less than 38°C for 2 to 6 hours (according to the viscosity) with stirring. The rate of agitation was reduced when working with high-viscosity polymers to avoid foaming of the mixture. Once a clear, lump-free mixture was formed, distilled water was used to adjust the final concentration.

Excessive bubble formation was observed in cellulose-based gels such as 10% (w/w) medium viscosity hydroxypropylcellulose (HPC-M) and 5% (w/w) high viscosity hydroxypropylcellulose (HPC-H), and HPMC 100 000. Entrapped air was removed by transferring them to 50-ml conical tubes and centrifuging at 3500 rpm for 5 minutes. However, HPMC MNs were not further studied because they caused skin irritation.

### Microfabrication of Swellable MN Arrays

To prepare the PVA-gelatin MN arrays, PVA and gelatin hydrogels were physically cross-linked by cryogelation. A total of 90 mg of the cross-linked mixture (20% w/v PVA and 10% w/v gelatin) was injected into the PDMS micromold as explained in the procedure of microfabrication of dissolvable MNs, but this time, the loaded micromolds were frozen at −20°C for 12 hours, followed by thawing at 25°C for 12 hours. This freeze-thaw cycle was repeated three times.

### Microfabrication of Biodegradable MN Arrays

To prepare the chitosan (medium molecular weight in the range of 190 000–300 000 with degree of deacetylation ≥75%) (Sigma-Aldrich, St.Louis, MO, USA) MN arrays, the required amount of chitosan were gradually added to half of the desired amount of glacial acetic acid. The remaining glacial acetic acid was added to yield a clear, lump-free solution with final concentration of 3% (w/w) chitosan that was then micromolded as in the above-mentioned procedure.

PLGA MNs were fabricated according to a previously published paper [36] with some modifications. The required amount of biodegradable PLGA pellets (lactide:glycolide 50∶50) (Sigma-Aldrich, St.Louis, MO, USA) was inserted into the PDMS micromolds using forceps, and the PDMS molds were gently placed in petri dishes, and exposed to −60 cmHg of vacuum pressure for 6 hours at 135°C (Vacuum Oven, Model OV-02; Jeio Tech Co. Ltd., Seoul, Korea). Created microbubbles during vacuum treatment were eliminated through several ventilation steps. The PDMS micromolds with melted PLGA were then carefully transferred to the refrigerator and allowed to cool and solidify for 15 minutes.

### Scanning Electron Microscopy (SEM) Analysis of MN Arrays

MN arrays were mounted on circular discs and morphologically characterized with an environmental scanning electron microscope (ESEM) in high-vacuum mode using the ETD detector at 10^−5^ Torr and 15 kV (FEI QuantaTM Environmental Scanning Electron Microscopes, Model Quanta 200 FEG; FEI, Oregon, USA). The specimens were first sputter-coated with an ion beam-based system (Precision Etching Coating System, PECS™, Model 682; Gatan Inc., Pleasanton, CA) that contained a single vacuum, and the layer thickness was controlled with a film thickness monitor (Film Thickness Monitor Controller, Model 681.20000; Gatan Inc., Pleasanton, CA). Computer software (XT Microscope Control; Version Quanta Oregon, USA) was used to display the SEM images. The magnification, tilt degree, spots, width and other imaging characteristics were reported on the SEM images.

### Differential Scanning Calorimetry (DSC) Analysis of MN Base

The glass to rubber transition of MN base materials, melting peak, and delta Cp were investigated using differential scanning calorimetry (DSC) system (Netzsch 204 F1 Phoenix®; Gerätebau GmbH, Wittelsbacherstraβe, Bavaria, Germany).

The sealed polymer film samples in 25-μl aluminum crucibles (Netzsch 100; Gerätebau GmbH, Bavaria, Germany) were heated at a linear heating rate of 10°C/min from 20 to 300°C, and nitrogen flow rates was 40 ml/min (purge gas MFC). Extrapolated onset, peak, and offset temperatures were calculated with Netzsch-compatible software (Proteus® Software for Thermal Analysis, Version 4.8.3. for Netzsch DSC 204 F1; Gerätebau GmbH, Bavaria, Germany).

### Mechanical Performance of Polymeric MN Arrays

Some of the polymeric MNs were tested for bending strength using a micromechanical test machine (Instron® Model 5969; Instron, Norwood, MA).

**Figure 4:**
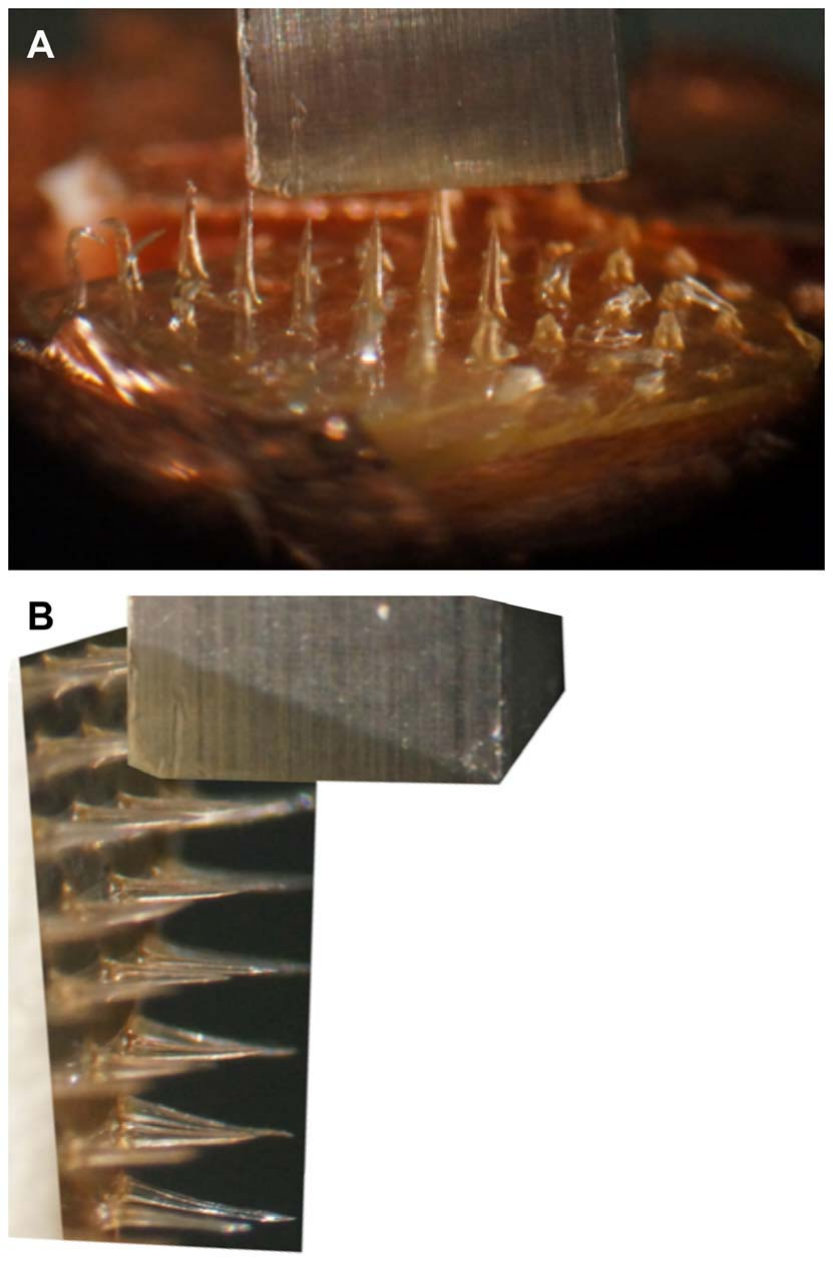
Micrographs of polymeric MN axial fracture and transverse fracture tests. (A) Digital photograph of SA MN pressed against the metal mill during axial fracture force measurement with the micromechanical tester (Instron® Model 5969; Instron, Norwood, MA). (B) MN shafts were transversely pressed against the metal mill for measurement of the transverse fracture force by way of the micromechanical tester (Instron® Model 5969, Instron, Norwood, MA).

Axial fracture forces (Fig. 4(A)) and transverse failure forces (Fig. 4(B)) of polymeric MNs were investigated to obtain the MN failure forces in a manner similar to that of an earlier study [10]. Data were analyzed with BlueHill 3 Testing Software for Mechanical Testing Systems (Instron, Norwood, MA).

### Measurement of Axial Fracture Force

Displacement versus force measurements was reported. A custom-made cuboidal metallic mill (length, 3 cm; cross-sectional area, 2 mm^2^) was fixed on the upper station, and MNs was mounted on the lower station of the micromechanical tester (Instron® Model 5969, Instron, Norwood, MA). A single MN was pressed into the metal mill at 500 µm/sec in each test (Fig. 4(A). The mill and MN were aligned using a microscope camera (Carl Zeiss AG, Jena, Germany). Upon maximum force application, the force either suddenly decreased or saturated. In the case of SA, the point before the sudden decrease was used as the needle failure force. In the case of PLGA, the point before the saturation point was accepted as the needle failure force. All needle failure forces were verified microscopically.

### Measurement of Transverse Fracture Force

Force versus displacement curves was obtained using a micromechanical tester (Instron® Model 5969; Instron, Norwood, MA). MNs were vertically attached to a square ABS plastic stub (cross-sectional area, 2 cm^2^) and fixed on the lower station, and a custom-made cuboidal metallic mill (length, 3 cm; cross-sectional area, 2 mm^2^) was attached to the upper station of the micromechanical tester. The MN array was carefully aligned with the aid of a microscope camera (Carl Zeiss AG, Jena, Germany) such that the metal mill could freely touch and fracture the single MN at a known height, where the test speed was 100 µm/sec (Fig. 4(B)). Upon maximum force application, the force suddenly decreased. The point before the sudden decrease was accepted as the needle failure force that was confirmed by microscopic evaluation.

### Statistical Analysis

The statistical comparisons of the practical measurements of height of various polymeric MNs were performed using a one-way analysis of variance (ANOVA), and *p<*0.05 was considered to indicate a statistically significant difference. Six different polymeric material groups were used with 216 values for each. Significant differences between Tg mid values of different polymeric MN bases were examined utilizing Kruskal-Wallis statistic.

## Results and Discussion

Master micro array templates can be fabricated by dry, wet anisotropic, X-ray, UV, and ion beam etching, excimer laser micromachining, laser drilling, hot embossing, and microinjection molding. However, most of these micro fabrication processes are slow and limited to few materials, and necessitate intricate course of actions [43]. Micromachining is a novel single step microfabrication method and can utilize many substrate types, such as PMMA (Poly methyl methacrylate), PLGA, aluminum alloys, stainless steel, ceramics, and metal sheets, can be used for fabricating miniaturized parts with it [21]–[23], [25].

Due to having low surface energy and abovementioned unique properties of PDMS, conventional micromolding was perfectly matched with micro milled pyramidal master needles for inverse replication of them. None of the six PDMS micromolds adhered to master templates. Theoretical measurements of the micromolds and actual measurements of the polymeric MNs were very close even after many uses of PDMS micromolds (Table 1).

**Table 1:** Summary of the physical features of polymeric MN arrays prepared from PDMS micromolds.

Polymeric MN Property	Polymeric MN Type	Height (µm)	Base diameter (µm)	MN inter base spacing (µm)	Aspect ratio
**Dissolvable**	SA (10% w/w)	857.25±7.35	206.70±2.94	497.96±3.53	4.15±0.07
	HPC-M (10% w/w)	877.27±6.20	247.81±1.70	497.00±1.59	3.54±0.04
	HPC-H (5% w/w)	897.23±3.80	249.38±1.99	495.89±2.22	3.60±0.03
**Swellable**	PVA-Gelatin (20∶10 w/v)	894.94±4.81	249.84±1.47	498.89±1.34	3.58±0.02
**Bio-degradable**	Chitosan (3% w/w)	826.89±15.09	248.24±2.62	481.14±4.56	3.33±0.06
	PLGA (50∶50)	899.89±1.66	250.39±1.12	499.18±1.12	3.59±0.02

Data are presented as the mean and SD (*n* = 216).

Figure 1 (A, B) shows a view of the top of a PDMS micromold, into which the polymeric materials flow after centrifugation or vacuuming. In Fig. 1(B), the distance between the microholes, indicated as the MN interbase spacing, was determined to be 500 µm. The MN base diameter was determined to be 250 µm. Both measurements were consistent with the physical dimensions of the actual polymeric MNs. Figure 1(C) shows a cross-sectional view, which was obtained by cutting the PDMS micromold vertically, and confirms the microholes as of pyramidal. Figure 1(D) shows a digital image of a 2×3 needle PDMS micromold.

Alkylation of pensile side chains of Si with CH_3_ (methyl) changes features of silicone, creates abovementioned unique physicochemical properties of PDMS (Figure 1 (E)), and increases its usage in medical devices [45], [46]. The physical characterization of pyramidal-shaped, out-of-plane SA, HPC-M, HPC-H, cross-linked PVA-gelatin, chitosan, and PLGA MNs (Table 1) were reported.

Although, the needles of each polymeric MNs were essentially the exact replicas of the master needles, the average height of the practically measured six different polymeric needles (n = 216) showed significant differences between different polymers with one way ANOVA (p<0.0001), which may have resulted from shape transitions during solidification, having different viscosities of distinct material composition.

Digital photographs of MN sections from 10×10 SA (Figure 2(A)), HPC-M (Figure 2(B)), HPC-H (Figure 2(C)), cross-linked PVA-gelatin (Figure 2(D)), chitosan (Figure 2(E)), and PLGA (Figure 2(F)) arrays fabricated from the six PDMS micromolds were illustrated. Figure 2(A) and Figure 2(F) revealed that relevant pyramidal needles were inflexible and rigid; the bases were regular, intact and smooth shaped. However, HPC MNs, cross-linked PVA-gelatin, and chitosan MNs were flexible.

Ratios of standard deviation to mean percentage of visualized needles were used to calculate the fabrication precision of polymeric MNs height, and inter base spacing using the Table 1 results. SA MNs could be fabricated within ±0.86% of the desired MN height. Furthermore, the precision of the base diameter was ±1.42%, and the precision of the interbase spacing was ±0.71%.

PLGA MNs could be fabricated within ±0.18% of the desired height. The fabrication precision of the base diameter was ±0.45%, and the precision of interbase spacing was ±0.22%. Other inversely fabricated polymeric MNs precision was found as excellent as the ones reported. In order to be concise we did not report the rest of the polymeric needles (HPC-M, HPC-H, PVA-gelatin, and Chitosan MN) replication precision, but could be calculated from the same way.

The precision of PLGA MN height was within ±0.18%, while it was within ±1.82% and ±0.86% for chitosan and SA MNs, respectively. Thus, PLGA MNs were better in terms of manufacturability, reliability, and repeatability. Another interesting finding was that the weakest material (chitosan) had a greater number of bent tips than the strongest material (biodegradable PLGA). In terms of height, PLGA MNs were 10 and 5 times more precise than chitosan MNs and SA MNs, respectively.

As it was declared in [22], [23], [25] this nano sensitive fabrication approach for generating metal master templates was very precise and accurate. Once we proof polymeric MNs physical robustness, this precision and accuracy will enable MNs, to be inserted into the skin uniformly without any bending. We have observed that this reduced the variability across tissue samples in drug delivery testing [41].

The structural morphology of the polymer SA, HPC-M, HPC-H, PVA-gelatin, and chitosan MNs was analyzed (Fig. 3(A)–(E)) by SEM. SEM measurements of polymeric MNs were in accordance with the theoretical geometries of the pyramidal master templates.

The MNs and base substrates were very smooth and clear. The needle tips were uniformly sharp for each needle in the array for all examined polymeric materials. The smoothness and reproducibility of the MNs were acceptable, although tip bending was observed in some polymers especially with chitosan MNs. None of the polymeric MNs entirely failed due to fracture of the substrate or tip prior to mechanical testing or visualization.

For the sodium alginate microneedle (SA MN) bases, an exothermic peak was observed at 44.3°C that represented crystallization of the amorphous material. Melting occurred at approximately 197.3°C, and recrystallization of amorphous material and crystals was observed at 230.4°C. The glass transition (Tg mid) occurred at approximately 139.25±0.04°C, with a delta Cp of 2.18±0.27 J/(g·K).

An exothermic peak was observed at 44.5°C that corresponded to the crystallization of the amorphous HPC-M MN base material. Melting occurred between 200 and 250°C. The recrystallization of amorphous material and crystals occurred between 150 and 200°C. The glass transition (Tg mid) occurred at approximately 211.87±1.46°C, with a delta Cp of 1.92±1.32 J/(g·K).

The cross-linked PVA-gelatin MN base exhibited an exothermic peak corresponding to pure crystals at approximately 44.3°C. The first melting point was observed at approximately 176.7°C, and a second melting point was observed above 200°C. The glass transition (Tg mid) occurred at approximately 122.93±8.14°C, with a delta Cp of 0.94±0.45 J/(g·K).

The chitosan MN base had an exothermic peak at approximately 45.6°C that resulted from the crystallization of amorphous material. Melting occurred at approximately 221.4°C. The glass transition (Tg mid) occurred at approximately 157.97±17.51°C, with a delta Cp of 7.34±6.26 J/(g·K).

Having Tg value over room temperature increased potentiality of the SA, HPC-M, PVA-gelatin, and chitosan polymer as to be convenient material for MN preparations. Glass transition mid values for SA, HPC-M, PVA-Gelatin, and Chitosan MN bases were found significant different, when non parametric Kruskal-Wallis statistic was performed (*p* 0.0205, *p<*0.05).

It is important to manufacture strong, reproducible microscale devices, especially in the case of polymeric MN arrays. For instance, dissolvable MNs must have sufficient strength to penetrate the skin, and their mechanical failure must be evaluated.

Weak mechanical behavior limits the usage of MN-based systems. Silicon is a commonly used material in microdevice fabrication; however, it is too brittle for use *in vivo* [48]. Etched silicon MN arrays are more useful as master templates for preparing polymeric MNs using conventional micromolding techniques [17].

The typical failure force rose from 0.06 to 0.32 N/needle for PLGA and PGA MN when the Young's modulus was rose from 1 GPa to 10 GPa, respectively. Needle failure was rose from 0.10 to 0.22 N/needle as the needle length increased from 700 to 1500 µm with constant tip (25 µm) and base (200 µm) diameters [10].

The safety margin, or fraction of the failure force to insertion force, decreased from 3.8 to 1.7 as the increasing of needle length for biodegradable PLGA MNs [10] similar results were observed with e-Sell 200 MN [49], where the forces required for MN insertion into the skin was order of magnitude less than the needle failure forces. Decrease in aspect ratio of e-Sell 200 MN was in tune with the increase of needle robustness, where they found two-photon polymerization and PDMS micromolding effective way for acrylate based MNs fabrication [49].

MNs often cannot be fully inserted into the skin because the non-uniform surface of the skin causes imprecise axial insertion that result in transverse bending of the needles [10]. Therefore, the measurement of transverse failure force is performed to predict MN bending behavior during skin insertion.

The transverse failure forces of PGA needles, with 100- and 200-µm base diameters with a constant tip diameter (25 µm) and length (1 mm), were 0.058±0.012 and 0.24±0.05 N, respectively [10].

0.1 N/needle, and 0.5 N/needle were the needle failure points for (600-µm needle height, 300-µm base width) conical CMC MNs, and PLA MNs, respectively. Pyramidal MNs made of different material compositions were rated for their robustness starting with the strongest one as, PLA, amylopectin, CMC/BSA (80∶20%), BSA, and CMC MNs. More interestingly, the combination of BSA and CMC exhibited a more robust microstructure than CMC alone [30].

Young's modulus and hardness of non-toxic Gantrez® AN-139 MN were noted as 6.56±0.56 GPa, 385.6±12.00 MPa, respectively, which indicates it as a stiffness microneedle base material [50].

Table 2 displays the MN failure force for two types of MNs obtained from axial loadings. Biodegradable PLGA (50∶50) MNs were used as positive controls for comparison with dissolvable 10% (w/w) SA MNs. According to the results in Table 2, PLGA (50∶50) MNs were stronger and more resistant to axial loading than 10% (w/w) SA MNs. For failure to occur, PLGA MNs required a force of 1.06±0.02 N/needle under axial loading, whereas SA only required a force of 0.18±0.05 N/needle. It can therefore be concluded that the fracture force of PLGA MNs is approximately 5 times greater than that of SA MNs [40].

**Table 2:** Material type effects on MN axial (*n* = 5) and transverse (*n* = 2) failures.

Test Type	MN Type	Force/Needle (N)
**Axial Failure**	PLGA (50∶50)	1.06±0.02
	SA (10% w/w)	0.18±0.05
**Transverse Failure**	PLGA (50∶50)	0.46±0.04
	SA (10% w/w)	0.04±0.02

Both data are represented as the mean and SD.

**Figure 5:**
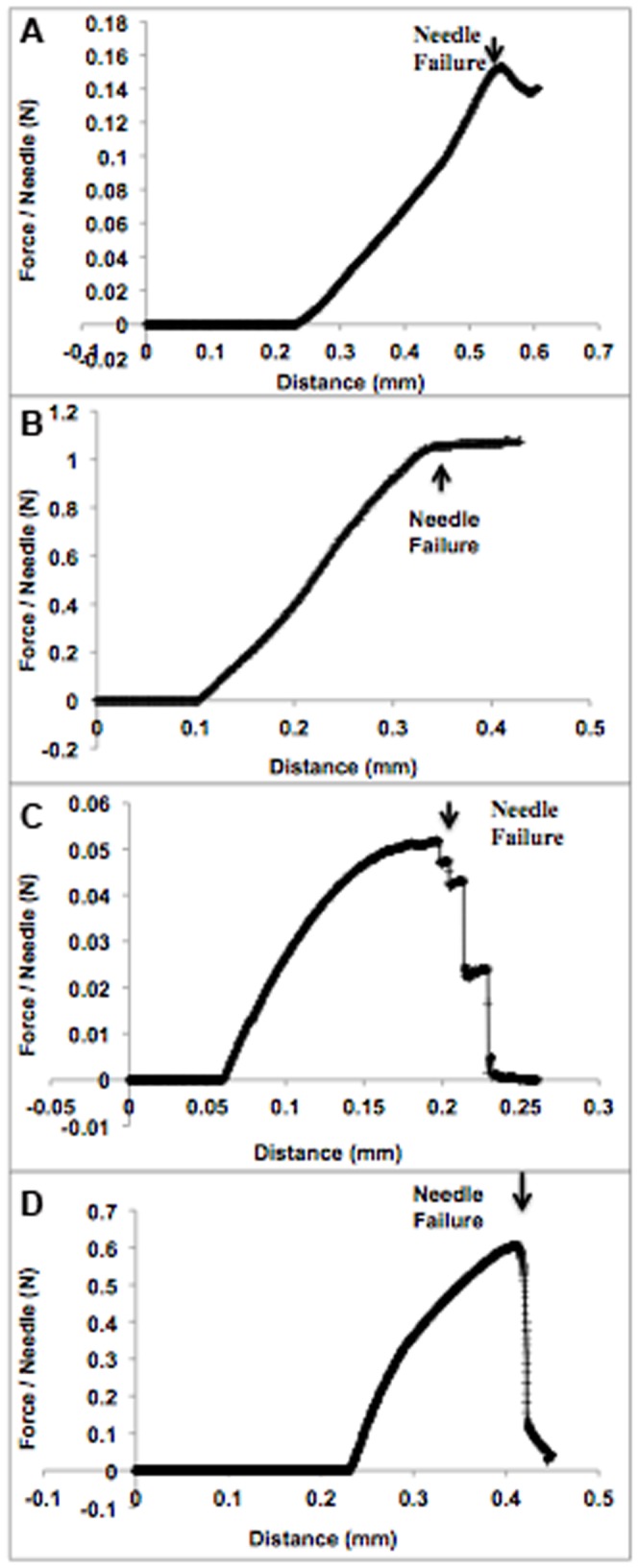
Results of mechanical analysis of SA and PLGA MN. (A) Mechanical analysis of polymeric 10% (w/w) SA MNs under axial loading. MN failure was interpreted as the sudden decrease in force. (B) Mechanical analysis of polymeric PLGA (50∶50) MNs under axial loading. MN failure was interpreted as the point at which the force became saturated. (C) Mechanical analysis of polymeric 10% (w/w) SA MNs under transverse loading. MN failure was interpreted as the sudden decrease in force. (D) Mechanical analysis of polymeric PLGA (50∶50) MNs under transverse loading. MN failure was interpreted as the sudden decrease in force

SA MN exhibited elastic deformation, as evident from the force versus displacement curve, which was linear for the axial compression test (force range between 0.0 and 0.18 N) (Fig. 5(A)). Similar deformation was observed with PLGA (50∶50) MNs, where the linear portion of the curve was between 0 and 1.06 N (Fig. 5(B)).

Table 2 displays transverse failure force data for two different polymeric MNs of the same geometry. Dissolvable 10% (w/w) SA MNs were compared with biodegradable PLGA (50∶50) MNs (positive control). An elastic deformation followed by plastic deformation was observed for both SA and PLGA MNs during transverse force tests (see Fig. 5(C) and (D)). According to the results in Table 2, biodegradable PLGA (50∶50) MNs were stronger than dissolvable 10% (w/w) SA MNs. PLGA MNs required a force of 0.46±0.04 N/needle to cause failure under transverse loading, whereas SA MNs only required a force of 0.04±0.02 N/needle.

## Conclusions

The micromilling and PDMS micromolding were effective methods to microfabricate solid, out-of-plane pyramidal needles high precisely and accurately with no limit to the substrate materials.

For the first time, pyramidal SA, chitosan, HPC-M, HPC-H, and cross-linked PVA-gelatin MNs polymers were inversely replicated with precisions ranging from ±0.18 to ±1.82% for height, ±0.45 to ±1.42% for base diameter, and ±0.22 to ±0.95% for interbase spacing [40].

Although biodegradable polymeric PLGA MNs have more resistance and mechanical stability, dissolvable SA MNs are proved to create microholes in the skin layers without breaking [41], since they have promising properties such as being hard/glassy at room temperature, representing relatively good deformation resistivity and mechanical robustness. SA MNs microfabricated in this work were evaluated in “poke and release” approach for enhancing protein delivery across the skin in our recent work [41].
